# Anæmia

**Published:** 1887-10

**Authors:** 


**Anæmia.** By Frederick P. Henry, M. D., Professor of Clinical Medicine in the Philadelphia Polyclinic, etc. Re-printed from the Polyclinic. Philadelphia: P. Blakiston, Son & Co., No. 1012 Walnut street. 1887.

This work on anæmia is the first treatise on this disease published in this contry. It discusses the varieties of anæmia as chlorosis, anæmia lymphatica, leuco-cythæmia, anæmia splenica, penicious anæmia, tox-anæmia, parasitic anæmia, etc. It is a valuable compend on a disease, or class of diseases, frequently met with by the practitioner. It is written in a clear and concise style, and shows the author to be an accurate student and possessor of a facile pen in recording the results of his observations.

